# Numerical Study into the Spanwise Effects for the Three-Dimensional Unsteady Flow over a Bio-Inspired Corrugated Infinite Wing at Low Reynolds Number

**DOI:** 10.3390/biomimetics11020090

**Published:** 2026-01-27

**Authors:** Almajd Alhinai, Torsten Schenkel

**Affiliations:** School of Engineering and Built Environment, Sheffield Hallam University, Howard Street, Sheffield S1 1WB, UK

**Keywords:** bio-inspired corrugated wing, low Reynolds number aerodynamics, computational fluid dynamics (CFD), flow separation and recirculation, lift and drag characteristics, angle of attack analysis

## Abstract

Corrugated insect wings inspire biomimetic aerodynamic design, yet their behaviour at low and transitional Reynolds numbers remains not fully understood. This study presents a three-dimensional computational analysis of flow over an infinite corrugated wing across Reynolds numbers from 10 to 10,000 and angles of attack from −5 to 20°, with emphasis on spanwise effects. An expanded verification and validation procedure ensured numerical reliability. At the lowest Reynolds numbers, the flow is steady and largely two-dimensional, with localised recirculation zones. As Reynolds numbers or angles of attack increase, the flow transitions to periodic vortex shedding, and three-dimensional structures appear. At a Reynolds number of ten thousand, periodic shedding occurs at zero degrees incidence, indicating a shift toward turbulent or bluff body-like behaviour. The examined corrugated profile does not exhibit a lift-to-drag benefit over smooth aerofoils in steady gliding, although root section corrugation helps delay separation in transitional regimes. This behaviour reflects mechanisms used by dragonflies to maintain stable gliding despite textured wings. By extending flow regime classification, the study identifies conditions where two-dimensional assumptions fail and highlights the influence of spanwise flow structures. These findings deepen understanding of insect wing aerodynamics and support biomimetic design of future wings.

## 1. Introduction

Bio-inspired corrugated aerofoils, modelled after insect wings such as those of dragonflies and cicadas, have drawn significant attention for their unique aerodynamic performance at low Reynolds numbers (typically $Re<10^{4}$) [1,2,3,4,5]. Unlike smooth aerofoils, which often suffer from early flow separation and low lift-to-drag (L/D) ratios in this regime [6], corrugated geometries can passively manipulate the flow to enhance lift and delay stall [7,8]. This phenomenon is critical for applications such as micro air vehicles (MAVs), where low-speed flight and manoeuvrability are essential. Despite extensive research into the flow over bio-inspired corrugated wings, several important uncertainties remain regarding their aerodynamic behaviour and underlying flow structures.

Early investigations into dragonfly gliding flight laid foundational insights into the aerodynamic role of wing corrugation at low Reynolds numbers. By conducting experiments in a small closed-circuit fluid flow channel at *Re* = 450, 800, and 900, Rees [9] demonstrated that insect wing sections, despite their corrugated structure, exhibit aerodynamic performance comparable to smooth aerofoils, with minimal penalties due to vortex formation. Rudolph [10] expanded on this by showing that corrugated wing sections delay stall and flow separation more effectively than flat plates, enhancing gliding stability. Azuma and Watanabe [11] combined experimental, analytical, and numerical methods and concluded that while corrugations offer limited aerodynamic benefit during gliding, they contribute significantly to structural stiffness required for flapping. Wakeling and Ellington [12] provided empirical evidence of dragonfly wings’ superior lift-to-drag ratios, highlighting their efficiency in steady gliding flight using video analysis, force measurements in a wind tunnel, and analytical methods to calculate lift and drag across *Re* = 700–2400. Finally, Kessel [13] confirmed that dragonfly wing profiles outperform technical aerofoils in aerodynamic efficiency, with vortices generated by corrugations smoothing airflow and maximising lift-to-drag ratios in wind tunnel experiments, making them a frequent reference in CFD studies. Collectively, these early investigations established both the aerodynamic and structural significance of wing corrugation, providing a foundation for subsequent experimental and computational studies.

Later research progressed through increasingly sophisticated experimental and computational techniques that directly connected these foundational findings to observed flow phenomena. Tamai et al. [14] and Hu et al. [15] used Particle Image Velocimetry (PIV) to directly observe unsteady vortex structures and flow separation around a corrugated wing profile derived from Kessel’s earlier work, confirming that corrugations actively suppress large-scale separation via momentum exchange and turbulence generation—mechanisms Kessel had previously inferred from steady-state data. Vargas et al. [16] employed 2D unsteady CFD simulations using Immersed Body Methods (IBM) to demonstrate that corrugated wings can produce lift comparable to or greater than smooth profiles, with recirculation zones reducing drag and improving gliding efficiency, noting that past experimental studies suggested negligible three-dimensional effects at low Reynolds numbers. Kim et al. [17] extended these insights by identifying specific corrugation geometries and valley locations that enhance lift through pressure field manipulation, complementing Tamai’s findings with parametric 2D unsteady CFD analysis, though these simulations neglected turbulence and spanwise 3D effects. Meng and Sun [18] addressed these limitations with 3D unsteady CFD, revealing that corrugation impacts vary with Reynolds number and geometry, promoting turbulence and improving performance at higher *Re*, while potentially hindering flow at lower *Re*, and highlighting spanwise variations in $C_{p}$ and separation bubbles that alter mean $C_{L}$ relative to 2D predictions. Finally, Barnes and Visbal [19] used high-fidelity 3D Implicit Large Eddy Simulations (ILES) to demonstrate that leading-edge corrugations trip the shear layer and promote early transition, reconciling earlier conflicting findings by showing that aerodynamic benefits emerge above *Re* ≈ 10,000 and that spanwise instabilities and finite-span effects play a critical role in lift and drag modulation. Their work further revealed that controlling leading-edge geometry can influence 3D transition pathways and force predictions, completing a more integrated understanding of corrugated wing aerodynamics.

Recent research has significantly advanced the understanding of dragonfly-inspired wing aerodynamics by integrating high-fidelity simulations, full 3D reconstructions, and nonlinear dynamical analysis. Chen and Skote [20] used 3D unsteady CFD with biologically accurate wing geometries to show that spanwise features such as nodus and vein taper stabilise flow and suppress oscillations seen in 2D models. While profiled wings often outperform corrugated ones at higher angles of attack, corrugation can still enhance aerodynamic efficiency at moderate Reynolds numbers and low angles by reducing viscous drag through trapped vortices. They also explain why experimental 3D wings did not show the strong oscillations of many 2D studies—realistic spanwise geometry (nodus, vein taper) produces stabilising spanwise flow. Bauerheim and Chopin [8] introduced a novel perspective by identifying a sudden route to aerodynamic chaos in a simplified 2D corrugated wing section. Their DNS results revealed that interactions between corrugations and a rear arc can trigger abrupt transitions to chaotic flow, with peak aerodynamic performance occurring near this bifurcation point. The authors report that the sudden transition to chaos appears in 2D DNS and persists in limited 3D checks, implying the identified bifurcation mechanism is robust but that fully realistic 3D spanwise morphology (as Chen and Skote emphasise) can modify or stabilise the unsteady pathway in practical wings. Chitsaz et al. [21] provided the first experimental PIV data on a full-scale, 3D-reconstructed dragonfly hindwing, confirming the presence of trapped vortices and delayed separation at low Reynolds numbers. Their work validated photogrammetry as a viable method for capturing fine corrugation details and highlighted the importance of spanwise flow effects. It also confirms the trapped vortex/“virtual profile” idea and delayed separation at low–moderate *Re* reported by Kesel and early lab work. Narita and Chiba [22] further advanced this line of inquiry by using CFD on a 3D-scanned hindwing of Pantala flavescens, demonstrating that the interaction between corrugation, vein structure, and global wing geometry (e.g., sweep and dihedral) governs the formation and aerodynamic role of valley recirculation zones. Narita moves beyond simple 2D “virtual-profile” arguments by showing that veins, taper and global orientation interact to produce three-dimensional flow structures that control integrated aerodynamic performance—so simple 2D explanations are necessary but not sufficient. Finally, Chiarini and Nastro [23] employed DNS to map the bifurcation landscape of a dragonfly-inspired aerofoil, identifying global modes and Floquet branches responsible for sudden transitions in aerodynamic behaviour. Their predictive framework links angle-of-attack and Reynolds number regimes to performance and stability, offering practical guidance for bioinspired wing design.

Although the literature shows that research initially focused on simplified two-dimensional geometries and has now progressed toward faithful three-dimensional reconstructions of dragonfly wings, the influence of three-dimensional spanwise effects is still not fully understood. A key reason is the lack of a standardised geometry approach. While most of the research focused on using the profiles from Kessel [13], for example [14,16,17], others used the simplified geometry from Newmann [24], e.g., [7,8,23]. In addition, most of the past studies on the wing corrugation claimed that 2-D studies were sufficient, as there was no velocity parallel to the wing span and no intrinsic three-dimensionality effects (e.g., [13,16,17,25]). However, Chen and Skote [20] have shown that the 2-D force and flow analyses neither reflect the three-dimensionality of the flow along the spanwise direction of the actual dragonfly wings nor accurately capture the overall performance of the corrugated wing. By comparing the time history of the lift coefficient of the 3D corrugated wing against the 2D corrugated profile from [17], the authors showed that the oscillation observed for the 2D simulations was suppressed when considering the full 3D wing, even at very high angles of attack. These observations highlight the need for fundamental understanding of the spanwise and wing aspect ratio effects on the flow over corrugated wings. Unlike flapping wings, where numerous studies have been reported on the effects of wing aspect ratio (see Baht et al. [26]), there are limited systematic studies for corrugated wings. Here we define the wing aspect ratio as the span divided by the chord length and note that the 3D studies reported here had a fixed aspect ratio that ranged from 1 for the DNS study of [23] to 8.83 for the unsteady simulations of [20].

In addition, experimental techniques such as Particle Image Velocimetry (PIV) are limited in their ability to resolve the flow within the smallest corrugation valleys, leaving finer-scale shear-layer dynamics unresolved. Given this limitation in experimental methods, the validation of numerical simulations becomes particularly challenging, as critical near-surface flow features and unsteady mechanisms cannot be fully captured for comparison. High-resolution numerical approaches, including Direct Numerical Simulation (DNS) and Large Eddy Simulation (LES), are therefore essential to complement experimental studies by resolving these small-scale features and providing deeper insight into the detailed flow physics. However, there remain significant numerical challenges, as highlighted in the review of Masud et al. [5]. For context, the finite wing simulations for Chen and Skote [20] required 3.45 million cells with a time step of $10^{-5}$, whereas the infinite wing of Chiarini and Nostro [23] required approximately 1.2 million spectral elements and approximately 260 million degrees of freedom. The scale and complexity of these simulations strongly suggest that parallel computing or access to powerful computational resources was likely involved. In addition, a major limitation in the current literature is the lack of a systematic approach to the verification and validation (V&V) of CFD models. Many studies rely primarily on grid-sensitivity analyses and benchmarking against available experimental data, which, while useful, do not provide a comprehensive framework for quantifying numerical and modelling uncertainties [27,28,29].

In this study, we present a numerical approach based on the Finite Element Method (FEM) that is run on a single workstation. The approach is based on the infinite wing arrangement used by [23]. Compared to the finite wing simulations of [20], this arrangement reduces the computational effort significantly while still retaining some of the key spanwise effects. The overall objective is to provide a fundamental understanding of the corrugation effects in the spanwise direction. In addition, our verification strategy will extend beyond traditional grid-independence studies to include the estimation of the Grid Convergence Index (GCI), the apparent order of discretisation, and the asymptotic range of convergence. Likewise, our validation approach will go beyond benchmarking CFD results against published data, incorporating targeted validation experiments to rigorously assess and demonstrate the predictive capabilities of our simulations.

## 2. Methodology

**Figure 1:**
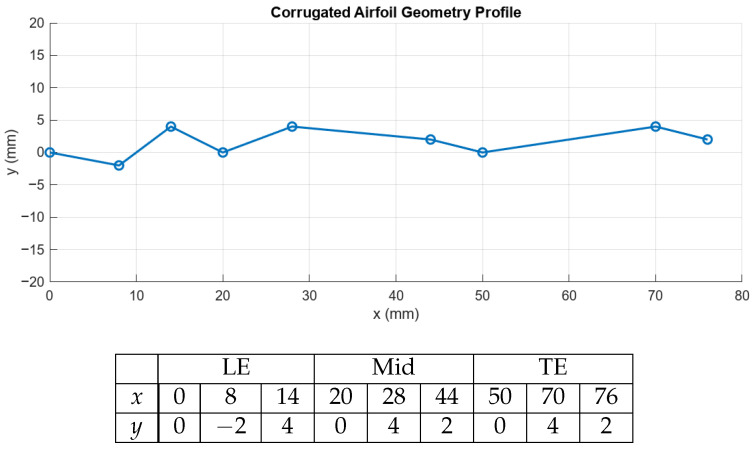
Corrugated aerofoil geometry profile coordinates in [mm], profile 1 from [13]. The thickness is 0.04× chord length. The points are grouped as leading edge (LE), midsection (Mid), and trailing edge (TE) and used as measurement points.

This study will consider the flow over the corrugated aerofoil from Kessel [13], profile 1 in particular, with minor adjustment to the thickness. Several studies have considered this profile, but limited research was performed for very low Reynolds numbers using 3D simulations. The methodology presented here is based on previous work [30] where we considered a 2D flow at Re = 10,000. The coordinate points for the profile are given in Figure 1.

### 2.1. Grid Generation and Numerical Method

The commercial Finite Element Method (FEM) software COMSOL Multiphysics (V 5.0) solves the following form of the unsteady Navier–Stokes equations:

(1) $$\rho \frac{\partial u}{\partial t}+\rho \left(u\cdot \nabla \right)u=\nabla \left[-pI+\mu \left(\nabla u+{\left(\nabla u\right)}^{T}\right)\right]+F$$

(2) ρ∇·u=0

**Figure 2:**
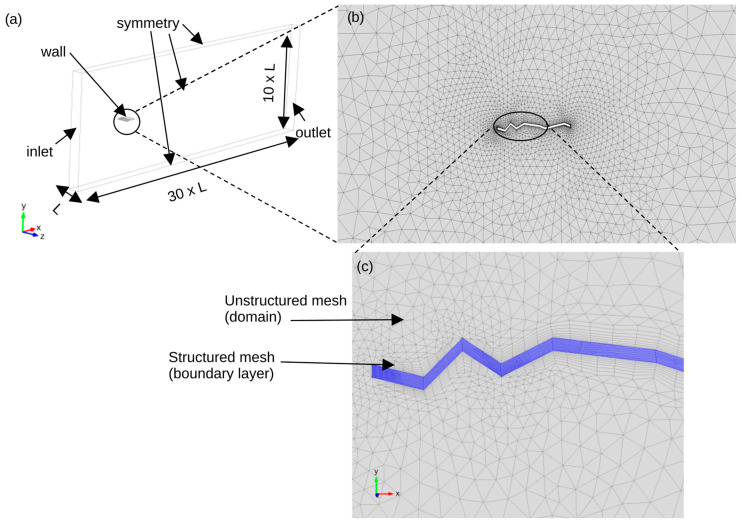
Domain and Grid. The domain size is 30× the chord length in the streamwise direction, 10× the chord length in the longitudinal direction, and 1× chord length in the spanwise direction as shown in (**a**). The mesh consists of 19,704 elements for illustration, as shown in (**b**). A close up of the mesh is shown in (**c**).

Full details on the solution of the Navier–Stokes equation using the FEM are given by [31,32]. Here the time-dependent laminar flow physics interface was used throughout. To obtain a sharper solution and remove the oscillations of the solution, the mesh needs to be refined locally at the boundary layers. Here we have to use a denser mesh near the walls where the boundary layer develops in order to satisfy the no-slip condition, as shown in Figure 2c. The boundary conditions used in this simulation (Figure 2a) are as follows:

- Velocity inlet: The velocity vector is applied as a Dirichlet condition: $u=-nu_{0}$, where n is the outward normal and $u_{0}$ the inflow speed.
- Pressure outlet: A zero-pressure Dirichlet condition is applied, $p=p_{0}=0$, assuming parallel flow with negligible viscous stress. The velocity satisfies the natural (Neumann) condition $\mu \left[\nabla u+{\left(\nabla u\right)}^{T}\right]\cdot n=0$, so the velocity gradient is not explicitly prescribed.
- Symmetry: Normal velocity is zero, u·n=0, while tangential stress vanishes, $\left[\mu \left(\nabla u+{\left(\nabla u\right)}^{T}\right)\cdot n\right]\cdot t=0$ (t is any tangential direction). This combines Dirichlet (normal velocity) and Neumann (tangential stress) conditions.
- Wall: The no-slip condition enforces u=0, ensuring the fluid adheres to the wall. This is a Dirichlet condition on all velocity components.

Additional simulation parameters are provided in Table 1. We note here that the *Re* studies were conducted by parametric sweeping using the dynamic viscosity. The value of the inlet velocity (1.3158 m/s) was used to match the Reynolds numbers with previous studies.

The parameters used to characterise the flow over the aerofoil are represented by the following Reynolds and Strouhal numbers in addition to the lift, drag, pressure, and skin friction coefficients, and their definitions are as follows:

(3) $$\begin{array}{c} Re=\frac{\rho uL_{c}}{\mu } \end{array}$$

(4) $$\begin{array}{c} St=\frac{L_{c}f_{dom}}{u} \end{array}$$

(5) $$\begin{array}{c} C_{l}=\frac{L}{0.5\rho u^{2}L_{c}^{2}} \end{array}$$

(6) $$\begin{array}{c} C_{d}=\frac{D}{0.5\rho u^{2}L_{c}^{2}} \end{array}$$

(7) $$\begin{array}{c} C_{p}=\frac{p}{0.5\rho u^{2}L_{c}} \end{array}$$

(8) $$\begin{array}{c} C_{f}=\frac{\tau_{w}}{0.5\rho u^{2}L_{c}} \end{array}$$

where $f_{dom}$ is the dominant frequency, *L* and *D* are the lift and drag forces, respectively, and $\tau_{w}$ is the wall shear stress.

A hybrid mesh approach was used to generate the grid, where quadrilateral elements were used around the no-slip condition to resolve the boundary layer and tetrahedral elements were used elsewhere, as shown in Figure 2. The 3D domain was constructed by extruding the 2D plane by one chord length (AR = 1) in the spanwise direction. A second-order discretisation scheme was used throughout the analysis. The total number of degrees of freedom for each grid is given in the verification section. The solution was obtained using the following sequence:

1. Generate an initial solution using the steady solver and make sure the pseudo time stepping option is activated.
2. Use the steady solution as the initial conditions for the unsteady solver.
3. Deactivate pseudo time stepping and run the unsteady solver for at least 3 s with a 0.02 s time step.

The time step is calculated based on the Courant condition,

(9) $$\Delta t\leq C\;\frac{\Delta x}{u}.$$

Here, we used C = 1, with an average grid spacing (Δx = 0.04 m) based on the largest element size and the velocity (*u*) = 1.3158 m/s.

All CFD simulations were conducted on a single workstation equipped with an Intel Core i7-1265H processor, 16 GB of RAM, and the Windows 11 operating system. A total of 32 simulations were considered, where the simulation times varied from a few minutes up to 12 h per run from the lowest to highest Reynolds number.

### 2.2. Verification

A grid convergence study was conducted using the Python package pyGCS [33], which is based on the procedures outlined in [34]. The case considered here is *AoA* = 0° and *Re* = 10,000 to allow for comparison with [13]. Here, the convergence study over four grids was conducted where ϕ represents the coefficient of lift, Table 2, or coefficient of drag, Table 3 and $\phi_{ext}$ are its extrapolated values. $N_{DoF}$ is the number of grid elements or degrees of freedom, *r* is the refinement ratio between two successive grids, GCI is the grid convergence index in percent and its asymptotic value is provided by $GCI_{asy}$, where a value close to unity indicates a grid convergence. The order achieved in the simulation is given by *p*, where the formal order used in the simulations was 2. Note that a mismatch between the observed and formal orders does not necessarily indicate divergence [34].

Overall, we observe better convergence of the drag coefficient compared to lift, as shown by the $GCI_{asy}$ being close to unity, *p* matching the discretisation order, and $\phi_{ext}$ being closer to the experimental value. The percentage difference between the various grids is in the range of 1–5% and the extrapolated value percentage error compared to the experiment of [13] is Cl=13.8% and Cd=3.08%. This is sufficient to confirm that grid independence and convergence have been achieved and demonstrate reasonable prediction of the aerodynamic forces compared to wind tunnel experiments.

### 2.3. Validation

Following the validation guidelines outlined by Oberkampf and Roy [28], we conducted a set of targeted numerical experiments to establish the reliability of the present CFD simulations. Specifically, we performed a Reynolds-number study to examine the sensitivity of the predicted aerodynamic coefficients to changes in flow regime, evaluated the Kolmogorov scaling hypothesis to verify the appropriate representation of turbulent energy across scales, and compared the simulated Strouhal number estimations with measurements obtained from controlled wind-tunnel experiments. Together, these validation steps provide a systematic basis for assessing the predictive capability of the simulations.

#### 2.3.1. Re-Effects

**Figure 3:**
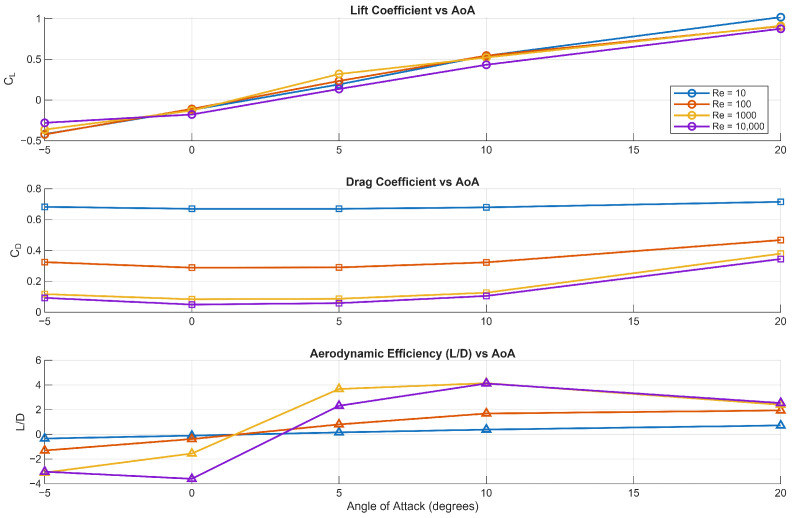
Variation in lift coefficient, drag coefficient, and aerodynamic efficiency with angle of attack for different Reynolds numbers.

In the Re-angle of attack study, shown in Figure 3, the following observations validate our simulations:

1. The lift coefficient is less sensitive to the Reynolds number when compared to the drag coefficient. Here we expect the lift coefficient to increase with increasing Reynolds number. However, the rate of increase diminishes as Re becomes higher, especially before reaching the transitional or fully turbulent regime.
2. A large drop in the drag coefficient is observed between Re = 10 and Re = 100. This is expected due to inertial effects becoming more dominant at higher Reynolds numbers as opposed to the domination of viscous effects at low Reynolds numbers.
3. The maximum L/D value is close to the value reported in the experiments of [12] (L/D = 4.6) for real dragonfly flights and the numerical simulation of [17,35].

#### 2.3.2. Energy Spectrum and Kologmorov Scaling

**Figure 4:**
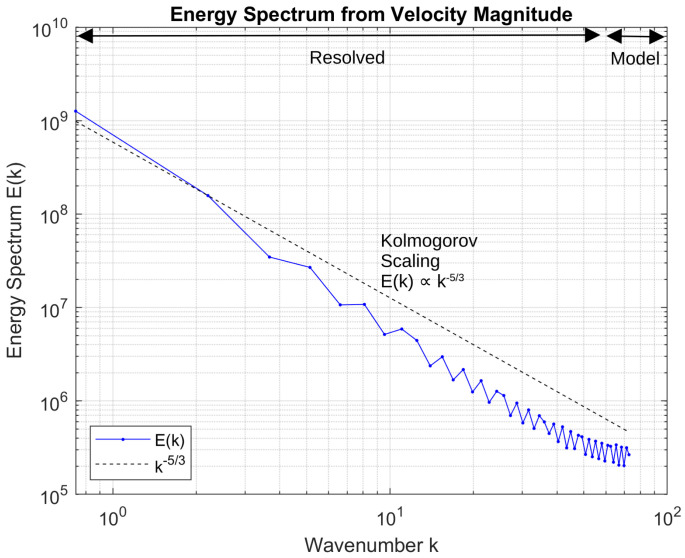
A plot of the energy spectrum against wave number for Re = 10,000 and *AoA* = 20°.

The Kolmogorov hypothesis predicts that in the inertial subrange of fully developed turbulence, the energy spectrum E(k) scales with the wavenumber k as $E\left(k\right)\propto k^{-5/3}$ [36]. Here, the velocity magnitude, given as $\sqrt{\left(u^{2}+v^{2}+w^{2}\right)}$, was used to plot the energy spectrum. Figure 4 presents the energy spectrum E(k) obtained from a three-dimensional simulation of an infinite wing at Reynolds number Re = 10,000. The spectrum exhibits a clear inertial subrange consistent with Kolmogorov scaling, $E\left(k\right)\propto k^{-5/3}$, indicating a physically plausible energy cascade from large to intermediate scales. The transition from the resolved region to the modelled region reflects the onset of numerical dissipation or subgrid-scale modelling, with the steep drop-off at high wavenumbers suggesting adequate resolution relative to the smallest turbulent scales. This spectral behaviour supports the fidelity of the simulation in capturing key features of wing-induced turbulence.

#### 2.3.3. Strouhal Number

The Strouhal number (St) comparison between wind tunnel experiments [37] and the current CFD simulations for an inclined flat plate is presented in Table 4. At an angle of attack (*AoA*) of 10°, the simulation underpredicts the experimental value by approximately 12.8%, potentially due to limitations in resolving near-wall unsteady structures or differences in turbulence modelling. In contrast, at 20°, the FEM result slightly overestimates the reference value by 4.42%, indicating improved agreement and suggesting that the simulation more accurately captures the dominant shedding dynamics at higher incidence. These results demonstrate reasonable consistency between numerical and experimental approaches, with deviations attributable to differences in flow conditions, geometric fidelity, and modelling assumptions.

**Figure 5:**
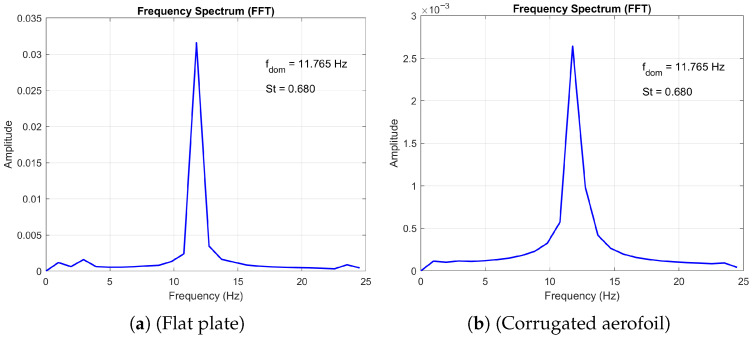
Comparison of the frequency spectrum plots at Re = 1000 and *AoA* = 10°.

**Figure 6:**
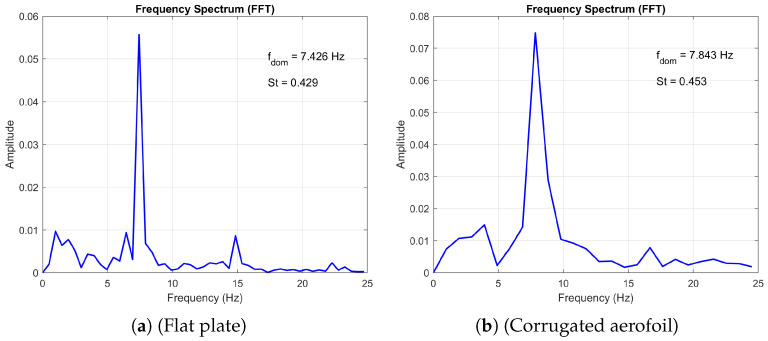
Comparison of the frequency spectrum plots at Re = 1000 and *AoA* = 20°.

Furthermore, the numerical study by Hord and Liang [25] reported that both the corrugated profile and the flat plate exhibit similar vortex shedding patterns at high angles of attack, behaving effectively as blunt bodies within the Reynolds number range of 500 to 2000. Our simulations corroborate this finding, as evidenced by the frequency spectrum plots for angles of attack of 10° and 20° (see Figure 5 and Figure 6, respectively) at Re=1000. Additionally, our results confirm the observed decrease in Strouhal number with increasing angle of attack as observed by [25,38].

## 3. Results and Discussion

This section presents the results beginning with methodological details and comparative analysis, followed by a comprehensive examination of aerodynamic behaviour across the three flow regimes defined by Chiariani and Nastro [23]. We first present the data-collection procedures, outlining the measurement protocols and processing methods used to collect the data. This is followed by a comparison with previous studies, allowing the present results to be situated within the broader context of existing literature. The remaining sections adopt the flow-regime classification proposed by Chiariani and Nastro [23], through which the observed aerodynamic behaviour is examined across three distinct regimes. Accordingly, we present and discuss the characteristics of steady, periodic, and non-periodic flows, emphasising the defining features and dynamical implications of each regime.

### 3.1. Data Collection

- Aerodynamic coefficients data was collected using surface integration of the reaction forces over the body. Here, a boundary probe was used to monitor the lift and drag forces at every time step.
- Point probe data of the velocity magnitude in the wake to measure the velocity autocorrelation function located at x = 1.1 $L_{c}$, y = 0, and z = 0.5 $L_{c}$.
- Point probe data for the pressure coefficient along the wing profile as shown in Figure 1 for both upper and lower surfaces.
- Line probe data of $C_{p}$ and skin friction $C_{f}$ distributions over the upper and lower surfaces.
- Strouhal number and dominant frequency plots using fast Fourier transform (FFT) on the lift coefficient signal.
- Flow visualisations using normalised vorticity, velocity streamlines, and other flow variables.

The results are presented based on the flow regimes identified by [23]. Here, we expand on this analysis by considering a wider Re and *AoA* range, as shown in Table 5.

### 3.2. Comparison with Previous Studies

Comparison with previous studies was conducted for various Reynolds numbers. The results for the aerodynamic coefficients at Re = 150 are given in Table 6. An angle of attack of 30° was selected for the comparison to confirm that the simulation is capable of capturing the periodic flow conditions. The comparison between the present three-dimensional simulation results and the two-dimensional reference data [17] indicates generally good agreement in aerodynamic performance, with some expected deviations. The lift coefficient exhibits a relatively small percentage difference of 2.83%, suggesting that the 3D simulation accurately captures the overall lifting characteristics of the wing. The drag coefficient, however, shows a larger deviation of 10.67%, which can be attributed to the additional three-dimensional effects, including induced drag and spanwise flow, that are inherently absent in 2D analyses. Consequently, the lift-to-drag ratio (L/D) differs by 9.29%, reflecting the combined influence of these 3D aerodynamic phenomena. These results demonstrate that, while the 3D simulation reproduces the primary aerodynamic trends observed in the 2D reference, the differences highlight the significance of three-dimensional flow effects, particularly in drag prediction. Overall, the level of agreement is satisfactory and consistent with expectations when extending from 2D to 3D modelling.

Next we have conducted an angle of attack study at Re = 1000 and compared the aerodynamic coefficients to the results from [25], as shown in Table 7. The comparison between the two-dimensional simulation results from [25] and our three-dimensional finite element method (FEM) simulations demonstrates consistent aerodynamic trends across the range of angles of attack analysed. The lift coefficient ($C_{l}$) shows decreasing percentage differences with increasing angle of attack, from approximately 9.63% at 5° to 4.23% at 20°, indicating improved correlation between the 2D and 3D models at higher angles where flow separation and three-dimensional effects may be less pronounced or better captured. Conversely, the drag coefficient ($C_{d}$) exhibits more substantial discrepancies, with the largest difference of 35% observed at 5°, gradually reducing to 10.74% at 20°. These differences primarily reflect the additional three-dimensional flow phenomena, including induced drag and spanwise flow effects, which are inherently absent in 2D simulations but accounted for in the 3D FEM approach. Moreover, differences in turbulence modelling and boundary conditions between the two simulation setups may further contribute to these variations. Despite these quantitative differences, the 3D FEM simulations successfully replicate the overall aerodynamic behaviour predicted by the 2D models, highlighting the importance of three-dimensional analysis for more accurate drag prediction and aerodynamic performance assessment.

**Figure 7:**
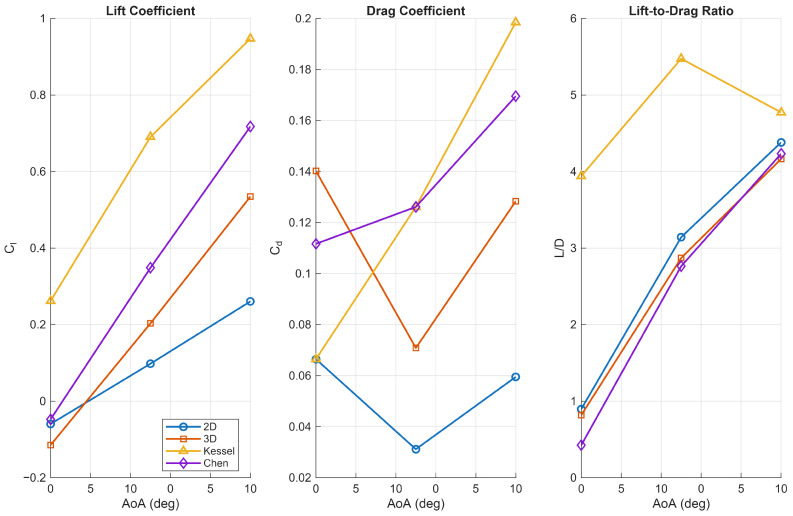
Comparison of the aerodynamic coefficients for the corrugated wing using 2D and 3D simulations, including the results from Kessel [13] and Chen and Skote [20].

Finally, the aerodynamic coefficients presented in Figure 7 compare the current two-dimensional (2D) and three-dimensional (3D) simulations with the three-dimensional simulation by Chen [20] and the wind tunnel experiments conducted by Kessel [13] for Re = 10,000. The lift coefficient ($C_{l}$) increased with angle of attack in all cases, with 3D simulations predicting higher lift than 2D. The 3D results closely matched Chen’s data but fell short of Kessel’s higher experimental values. Chen has noted discrepancies between Kessel’s experiments and 3D CFD results, citing experimental uncertainties and modelling limitations as potential causes. The 2D simulations consistently underestimated lift, highlighting their limited accuracy for this geometry. These results demonstrate that while 3D simulations improve lift predictions, further model refinement is necessary to fully capture the lift levels observed experimentally by Kessel.

Regarding the drag coefficient ($C_{d}$), the 2D simulation predicts the lowest drag values, reflecting the limitations of two-dimensional assumptions that exclude three-dimensional drag components such as induced drag. The current 3D simulation aligns well with Chen’s numerical results, both capturing the increasing drag trend observed experimentally. The elevated drag levels in Kessel’s wind tunnel measurements are likely influenced by physical effects such as surface roughness, support interference, and other experimental uncertainties not accounted for in numerical simulations.

The lift-to-drag ratio (L/D) further highlights these distinctions. While the 2D simulation suggests superior aerodynamic efficiency, the 3D simulation data reveal the performance penalties associated with three-dimensional flow phenomena. The current 3D simulation underpredicts the L/D ratio relative to Kessel’s measurements but remains in close agreement with Chen’s results, indicating consistency between independent 3D modelling approaches.

Overall, the comparison confirms that the current 3D simulations provide a reliable approximation of realistic aerodynamic behaviour, closely matching Chen’s reference data and capturing key three-dimensional flow effects. In contrast, 2D simulations consistently underpredict lift, drag, and lift-to-drag ratios, highlighting their limitations in representing the full aerodynamic efficiency of corrugated wings. The discrepancies with Kessel’s experimental data further underscore the influence of complex flow phenomena and the need for continued refinement in computational modelling to fully replicate observed performance.

### 3.3. Steady Flow

**Figure 8:**
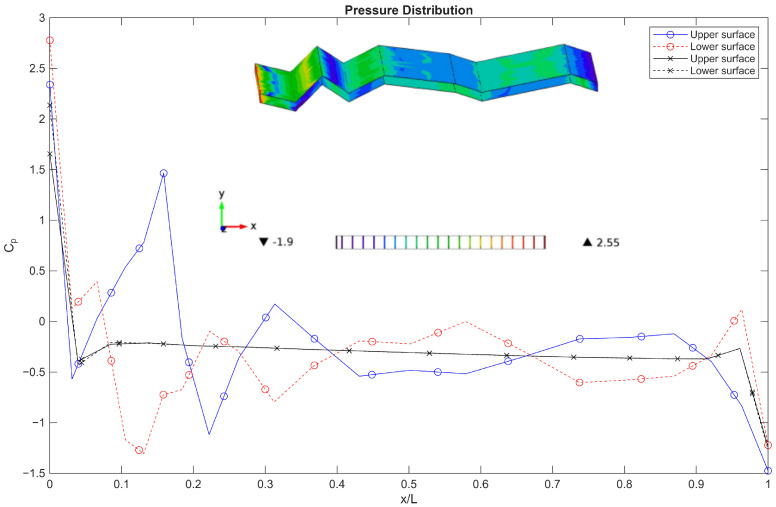
Comparison of the pressure distribution over a flat plate (black line) and the corrugated wing (blue and red) at Re below 100 and *AoA* = 0°. The surface plot (inset) shows the spanwise distribution over the upper surface.

**Figure 9:**
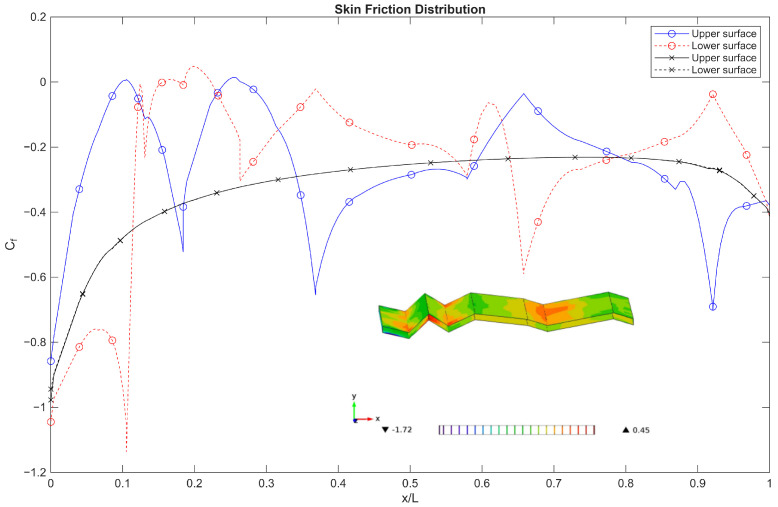
Comparison of the skin friction distribution over a flat plate (black line) and the corrugated wing (blue and red) at Re below 100 and *AoA* = 0°. The surface plot (inset) shows the spanwise distribution over the upper surface.

For Re = 10 and 100, the flow is generally steady for both a flat plate and corrugated aerofoil, where viscous effects dominate the flow as suggested by the high value of the drag coefficient in Figure 3. However, the distributions of pressure coefficient $\left(C_{p}\right)$ and skin-friction coefficient $\left(C_{f}\right)$, see Figure 8 and Figure 9, in the chordwise direction highlight clear differences between the flat plate and the corrugated aerofoil. As expected, the flat plate exhibits no net pressure difference along its chord, resulting in zero lift generation and an absence of any flow recirculation zones. In contrast, the corrugated aerofoil shows distinct localised separation and recirculation regions, evidenced by pronounced variations in both ($C_{p}$) and ($C_{f}$) along the chordwise direction. These features arise from the geometric undulations, which promote alternating zones of accelerated and decelerated flow.

Surface plots of the spanwise distributions (inset Figure 8 and Figure 9) further emphasise the inherently three-dimensional nature of the flow over the corrugated aerofoil. Unlike the flat plate, the corrugated configuration exhibits strongly non-uniform spanwise behaviour, even though the nominal simulations are two-dimensional. At Reynolds numbers below 100, such spanwise non-uniformities in ($C_{p}$) and ($C_{f}$) indicate that significant three-dimensional flow structures—such as secondary flows, and localised separation—play a dominant role in defining the aerodynamic loading. These variations imply that the pressure field, and therefore aerodynamic forces, can vary substantially along the span, which is particularly consequential in this ultra-low-Reynolds-number regime.

**Figure 10:**
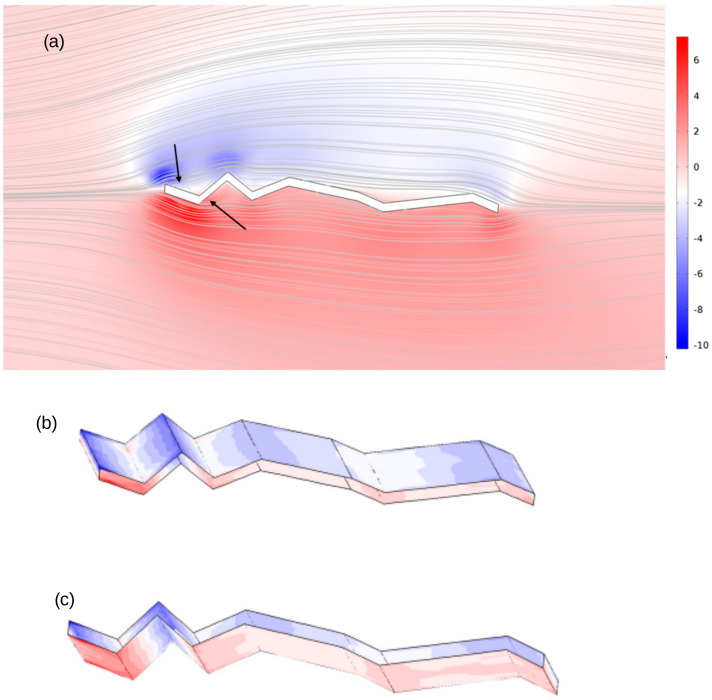
Normalised vorticity and velocity streamlines at *AoA* = 5° and Re is below 100. Arrows in (**a**) indicates the local recirculation regions. The spanwise distribution along the surface is shown in (**b**) for the upper surface and (**c**) for the lower surface.

This is further demonstrated for a small angle of attack (5°) using the normalised vorticity and velocity streamline plots shown in Figure 10, where the arrows indicate the localised flow separation and reattachment at the leading edge. The normalised vorticity fields demonstrate the aerodynamic impact of the corrugated wing geometry. The side view (Figure 10a) shows alternating regions of positive and negative vorticity aligned with the corrugations, indicating shear-layer formation and localised recirculation. On the top surface (Figure 10b), vorticity concentrations occur along ridges and leading-edge corrugations, consistent with flow acceleration and detachment. The lower surface (Figure 10c) exhibits complementary vorticity structures within troughs and trailing regions, reflecting reattachment and vortex pairing. These results confirm that the corrugated morphology promotes enhanced vortex generation and modifies flow symmetry, with measurable implications for lift and drag characteristics.

Collectively, these observations demonstrate the limitations of purely two-dimensional studies, even at very low Reynolds numbers where 2D assumptions are often presumed adequate. The presence of inherently three-dimensional flow phenomena suggests that accurate prediction of aerodynamic performance for corrugated aerofoils requires full three-dimensional analysis.

**Figure 11:**
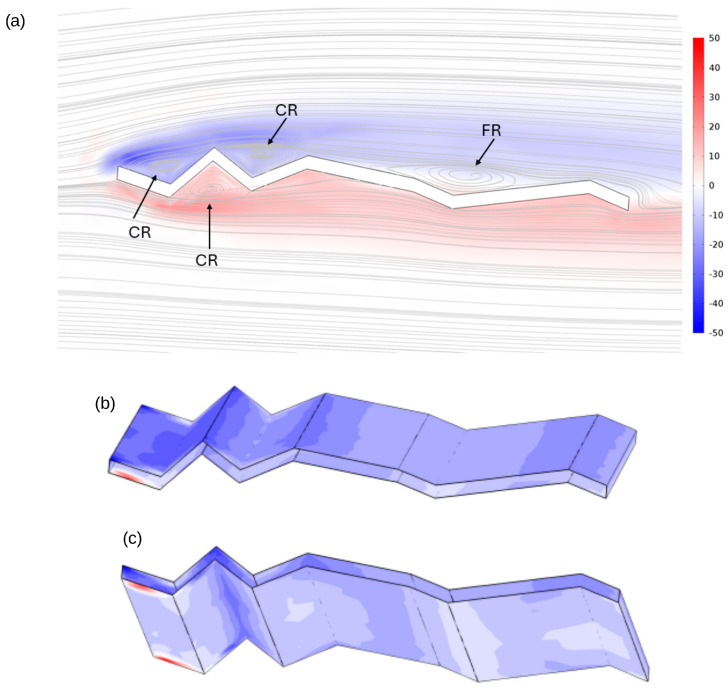
Normalised vorticity and velocity streamlines at *AoA* = 5° and Re = 1000. Arrows in (**a**) indicate the closed (CR) and free (FR) recirculation regions. The spanwise vorticity distribution along the surface is shown in (**b**) for the upper surface and (**c**) for the lower surface.

At an angle of attack of 5° and a Reynolds number of 1000, the normalised vorticity and streamline patterns reveal distinct flow regimes around the corrugated wing. Figure 11a identifies closed (CR) and free (FR) recirculation zones, with alternating vorticity bands aligned with the surface corrugations, indicating shear-layer formation and localised recirculation. The upper surface distribution (Figure 11b) shows concentrated vorticity along leading-edge ridges and spanwise undulations, consistent with flow detachment and rotational intensification. The lower surface (Figure 11c) exhibits complementary vorticity structures within troughs and trailing regions, suggesting reattachment and vortex pairing. These results confirm that the corrugated geometry induces spanwise variation in vorticity and promotes organised vortex structures that influence aerodynamic performance.

**Figure 12:**
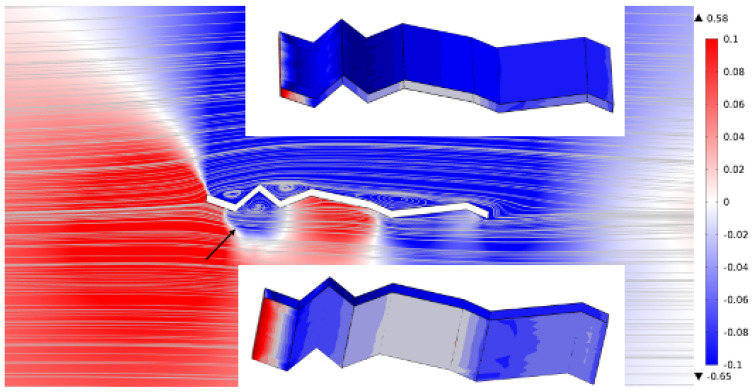
Pressure contour plot at *AoA* = 5° and Re = 1000 with velocity streamlines. The arrow indicated the favourable pressure region. Surface plots (inset) show the spanwise pressure distribution.

The pressure contour plot at an angle of attack of $5^{\circ }$ and Reynolds number of 1000 reveals distinct pressure gradients and streamline behaviour around the corrugated wing. Figure 12 shows a region of favourable pressure near the leading-edge corrugations, as indicated by the arrow, where streamlines converge and pressure decreases. The spanwise surface plots (Figure 12, inset) demonstrate non-uniform pressure distribution, with elevated pressure zones aligned with ridge crests and reduced pressure within troughs. These results confirm that the corrugated geometry induces localised pressure recovery and spanwise modulation, contributing to aerodynamic stability and potential lift enhancement.

### 3.4. Periodic Flow

The periodic flow emerges from a steady laminar base flow when the Reynolds number exceeds a critical value, triggering a Hopf bifurcation [23]. The 2D periodic regime is identified by a single peak in the frequency spectrum of the velocity signal, as shown in Figure 5. The frequency spectrum plots compare the vortex shedding characteristics of a flat plate and a corrugated aerofoil at Reynolds number Re=1000 and angle of attack $AoA=10^{\circ }$. Both surfaces exhibit a dominant peak at f=11.765Hz, corresponding to a Strouhal number of St=0.680, indicating similar shedding frequency. However, the amplitude of the corrugated aerofoil’s spectrum is notably lower than that of the flat plate, suggesting reduced vortex strength or energy content in the wake. This implies that the corrugated geometry may dampen flow instabilities or alter wake dynamics compared to the flat plate.

This regime persists until a secondary bifurcation occurs, which may lead to quasi-periodic, subharmonic, or three-dimensional flow depending on the angle of attack and Reynolds number. Figure 6 presents a comparative analysis of the frequency spectra obtained from the flat-plate and corrugated-aerofoil configurations at Re=1000 and an angle of attack of $20^{\circ }$. The flat plate exhibits a dominant shedding frequency of $f_{\mathrm{dom}}=7.428\;\mathrm{Hz}$, corresponding to a Strouhal number of St=0.429, while the corrugated aerofoil shows a slightly elevated peak at $f_{\mathrm{dom}}=7.843\;\mathrm{Hz}$ and St=0.453. This upward shift in frequency and Strouhal number suggests that the corrugated geometry modifies the wake dynamics, potentially enhancing vortex formation or delaying separation. Both spectra display sharp peaks indicative of coherent shedding; however, the aerofoil’s broader spectral energy distribution may reflect increased flow complexity or secondary instabilities introduced by surface corrugation. These findings highlight the sensitivity of vortex shedding characteristics to surface topology and underscore the aerodynamic influence of passive geometric modifications.

#### 3.4.1. 1D Velocity Correlation

**Figure 13:**
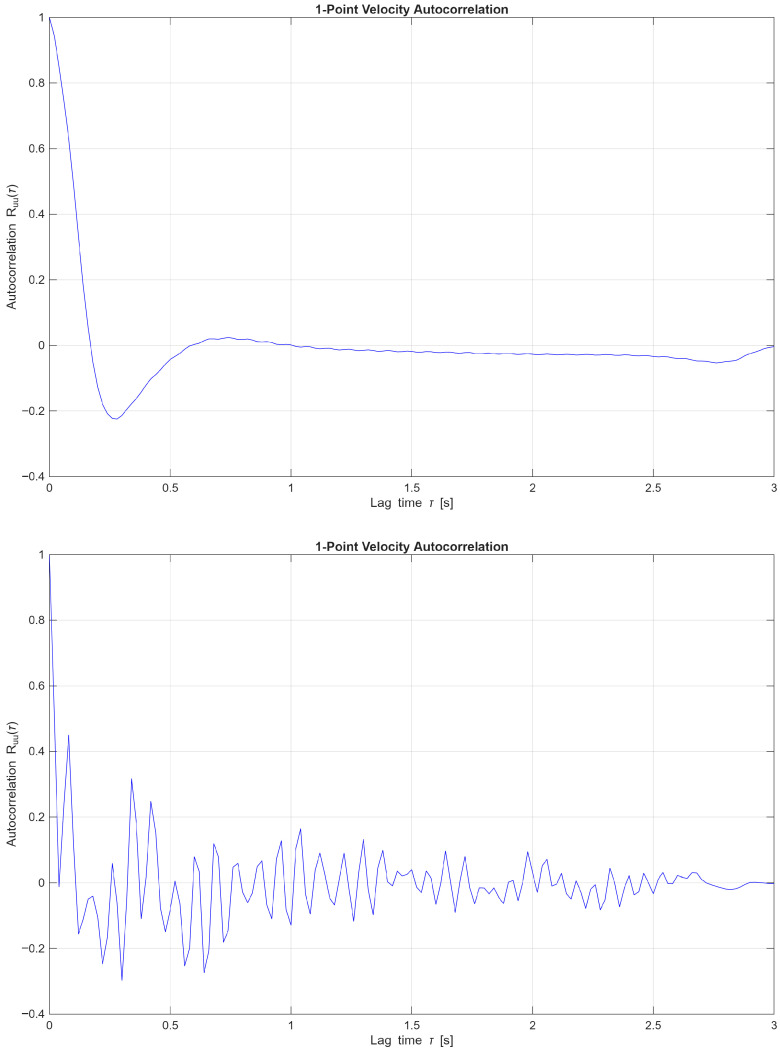
One-point velocity correlation at *AoA* = 10° and Re = 1000 [**upper**], 2D periodic flow and Re = 10,000 [**lower**], 3D non-periodic flow.

**Figure 14:**
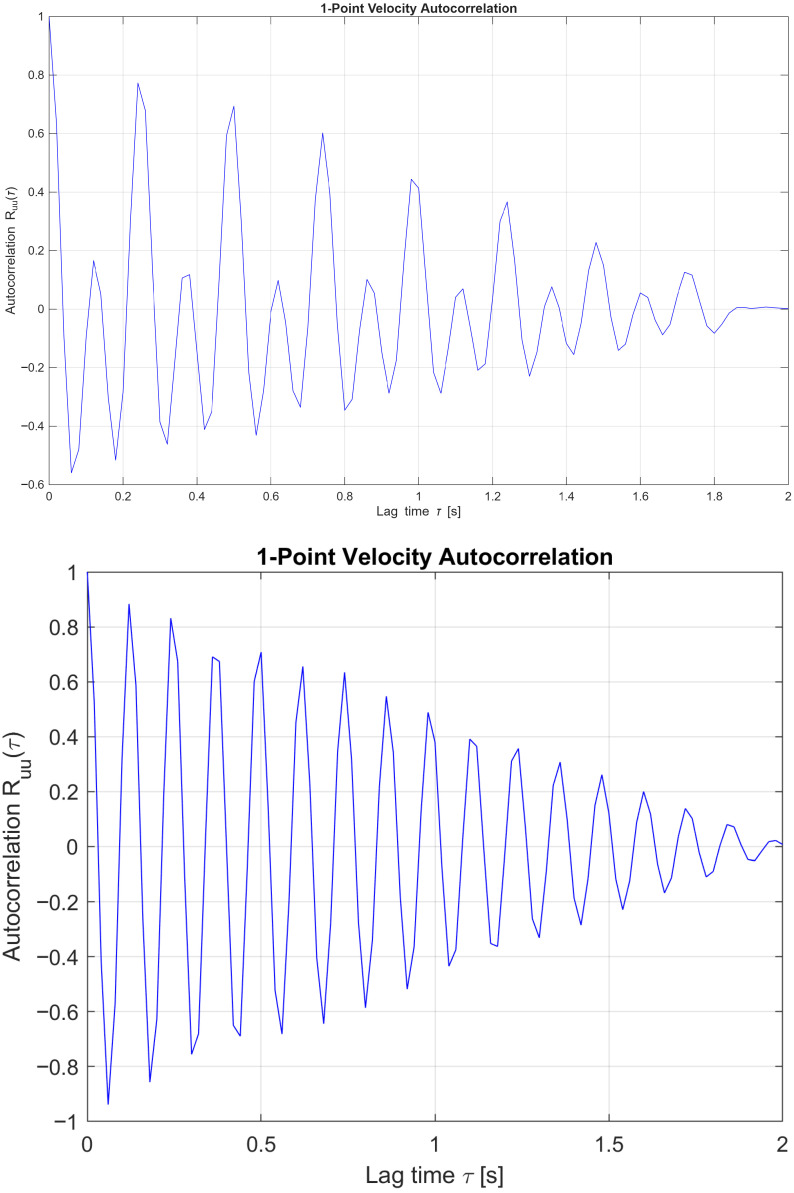
One-point velocity correlation at *AoA* = 20° and Re = 1000 [**upper**], and Re = 10,000 [**lower**], both in the 3D periodic flow regime.

To further examine the differences between the two-dimensional and three-dimensional periodic flow regimes, velocity-magnitude point-probe autocorrelation functions were compared at angles of attack of $10^{\circ }$ (Figure 13) and $20^{\circ }$ (Figure 14). The rate at which the autocorrelation function decays indicates how long velocity fluctuations remain correlated, providing insight into turbulence memory and mixing behaviour. Here, the one-point velocity correlation is given as

(10) $$\begin{array}{c} R_{ii}\left(\tau \right)=\langle u_{i}\left(t\right)\;u_{i}\left(t+\tau \right)\rangle \end{array}$$

This is the specific form of the temporal one-point autocorrelation function for the i-th velocity component at a fixed spatial point within the wake region. Here $R_{ii}\left(\tau \right)$ is a one-point, one-component, temporal autocorrelation function. $u_{i}\left(t\right)$ is the instantaneous velocity component in direction i and time t. $u_{i}\left(t+\tau \right)$ is the same component at a later time t+τ. The brackets 〈.〉 are the ensemble average. Finally, τ is the time lag.

Figure 13 presents the one-point velocity autocorrelation $R_{u}\left(\tau \right)$ for flows at an angle of attack of $10^{\circ }$, comparing a two-dimensional periodic case at Re=1000 with a three-dimensional non-periodic case at Re = 10,000. The 2D flow exhibits a rapid decay in autocorrelation with a smooth transition into negative values, characteristic of coherent, periodic vortex shedding with relatively short temporal memory. In contrast, the 3D flow displays sustained oscillations and a more gradual decay, indicative of complex, non-periodic dynamics involving intermittent structures and extended temporal correlations. These trends underscore the transition from organised, laminar-like shedding to turbulence-dominated, multi-scale interactions as the Reynolds number increases.

Figure 14 presents the one-point velocity autocorrelation at an angle of attack of $20^{\circ }$ for flows at Re=1000 and Re = 10,000, both of which lie within the three-dimensional periodic flow regime. At Re=1000, the autocorrelation exhibits a sustained, oscillatory decay, indicative of coherent, long-lived flow structures and quasi-periodic behaviour. In contrast, the Re = 10,000 case shows a more rapid decay accompanied by higher-frequency oscillations, reflecting reduced temporal coherence and elevated turbulence intensity. These comparative trends highlight the progression from organised vortex shedding toward increasingly chaotic dynamics as the Reynolds number increases, consistent with the onset of fully three-dimensional turbulent flow.

#### 3.4.2. Pressure Point Data

**Figure 15:**
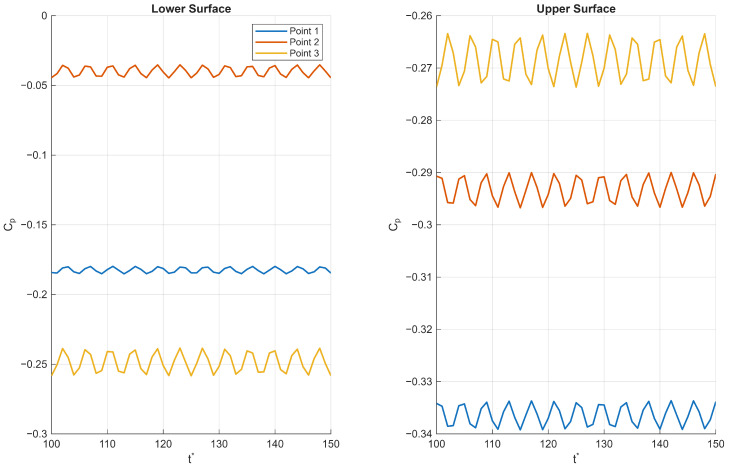
Pressure fluctuations at the trailing edge of a corrugated wing at *AoA* = 10° and Re = 1000, where t* is the non-dimensional time-step.

The pressure fluctuation patterns observed near both the leading and trailing edges of the corrugated wing reveal key aspects of the complex flow physics governing its aerodynamic behaviour. First we consider the trailing edge for the Re = 1000 and *AoA* = 10° case where the onset of unsteadiness was observed along the wing surface (Figure 15); the lower surface exhibits a pressure increase followed by a sharp drop, with unsteadiness intensifying downstream. This suggests the presence of flow separation or localised vortex shedding near point 3, which generates higher pressure fluctuations and reduced mean pressure. The upper surface shows a consistent pressure increase with similar unsteady patterns but maintains negative pressure coefficients overall, indicative of suction effects driving lift generation. The increasing fluctuation amplitude towards the trailing edge reflects the development of turbulent wake structures and flow instabilities common in bluff or corrugated geometries.

**Figure 16:**
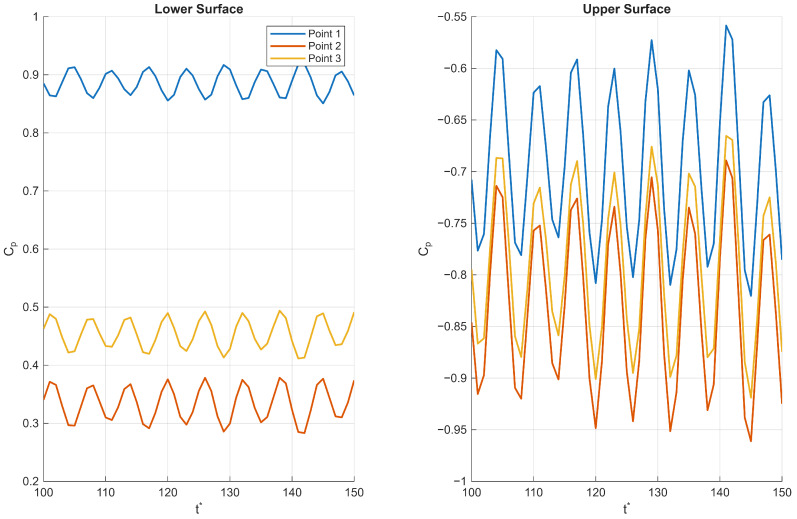
Pressure fluctuations at the leading edge of a corrugated wing at *AoA* = 20° and Re = 1000, where t* is the non-dimensional time-step.

Second, we consider the *AoA* = 20° case, where unsteadiness was observed throughout the profile; at the leading edge (Figure 16), the lower surface maintains positive pressure coefficients but with a non-monotonic spatial distribution—a decrease to a minimum at point 2 followed by an increase—suggesting a local pressure trough possibly caused by flow acceleration and separation bubbles formed around the corrugation peaks. The upper surface shows strong suction (negative pressure coefficient) with large fluctuations, implying that unsteady separation and reattachment phenomena dominate near the leading edge. The large amplitude fluctuations highlight the sensitivity of the flow to small disturbances in this region, which strongly influence overall aerodynamic performance.

Together, these observations reflect a highly three-dimensional, unsteady flow environment characterised by alternating zones of acceleration, separation, and vortex shedding that are inherent to corrugated wing profiles. The interplay of positive and negative pressures on the lower and upper surfaces, coupled with the spatial and temporal variations in pressure fluctuations, illustrates how the corrugated geometry modulates the boundary layer development and wake dynamics. This complex flow behaviour ultimately impacts lift, drag, and stability characteristics.

#### 3.4.3. Quasi 2D Periodic Flow

**Figure 17:**
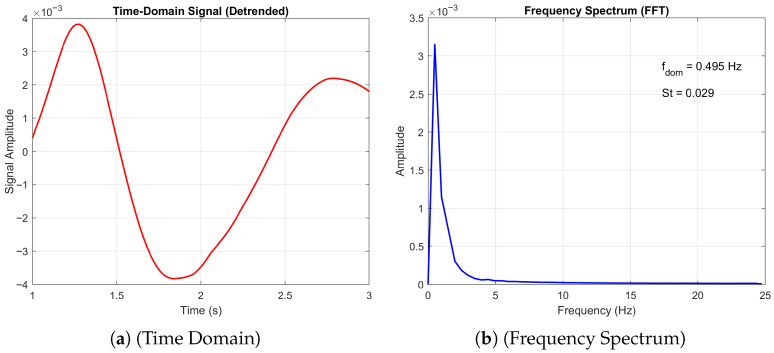
Time domain and frequency spectrum plots of the corrugated wing time domain and frequency plots at Re = 10,000 and *AoA* = 0°.

Finally, we consider the periodic flow at Re = 10,000. At an angle of attack = 0, the flow is quasi-2D periodic, as demonstrated by the long frequency period and low Strouhal number, as shown in Figure 17. Compared to a flat plate where no normal force is produced due to the symmetry of the geometry, the corrugated profile produces small periodic oscillations between lift and downforce. A comparison of the averaged values, see Figure 7, reveals that our results agree with the 3D simulations of [20] and highlights a discrepancy with the experimental data of [13]. This observation underscores the inherent challenges in characterising the transitional Reynolds-number regime within this flow configuration.

**Figure 18:**
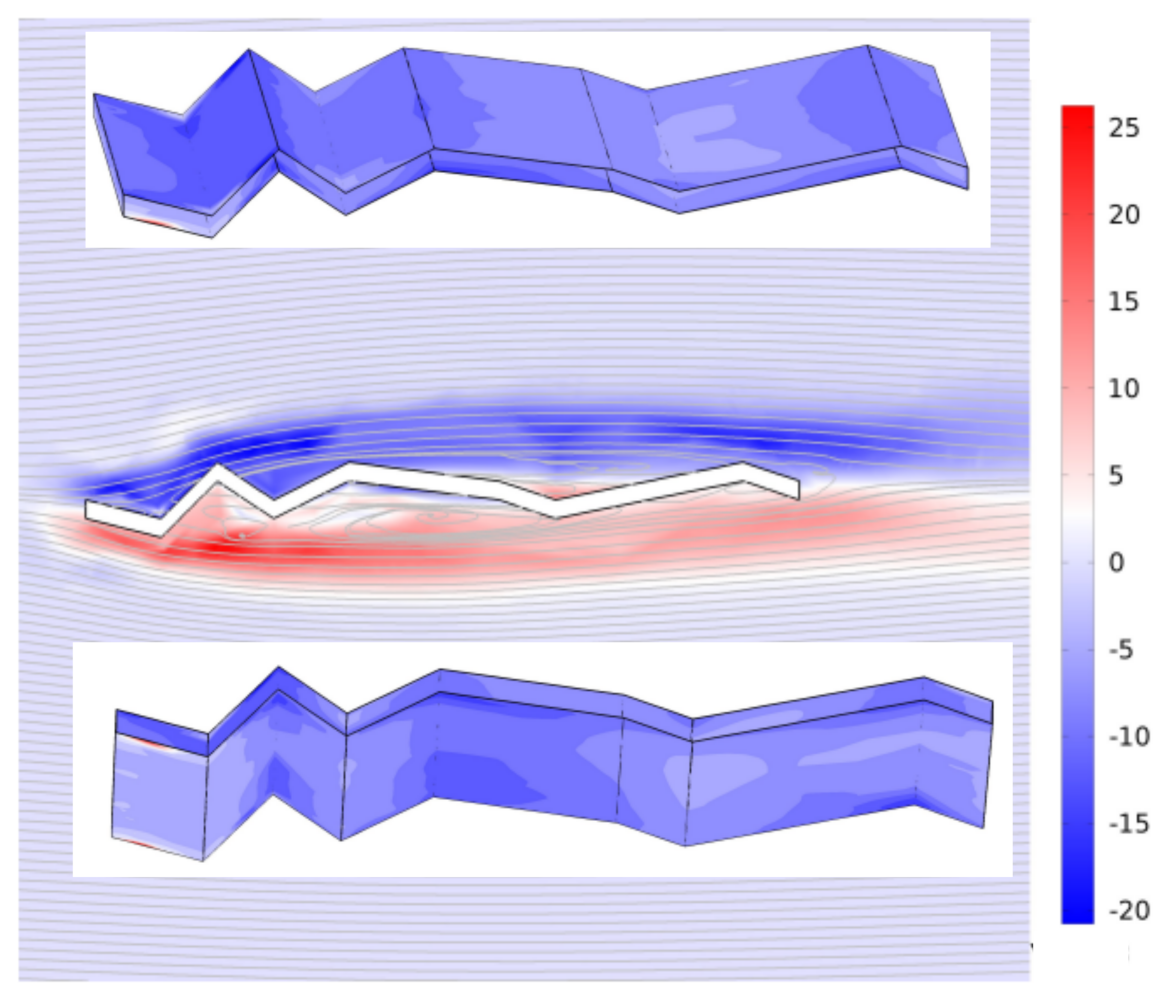
Normalised vorticity and velocity streamlines at *AoA* = 0° and Re = 10,000. Surface plots (inset) show the spanwise vorticity distribution.

Figure 18 shows the normalised vorticity field and velocity streamlines around the corrugated wing at zero angle of attack and Re = 10,000. The streamlines reveal complex flow interactions driven by geometric discontinuities. Upstream of the leading edge, streamlines remain uniform and evenly spaced, indicating steady inflow conditions. Upon encountering the corrugated geometry, the flow undergoes pronounced separation at each sharp edge, generating alternating vortices and recirculation zones downstream. These vortical structures are accompanied by curved shear layers and localised regions of high vorticity. The persistent distortion of streamlines in the downstream wake reflects sustained turbulence and energy dissipation, characteristic of bluff body flows with significant drag. The insets of Figure 18 present a normalised vorticity surface plot showing the spanwise direction, and revealing the three-dimensional structure of vortical features induced by the geometry. The surface exhibits alternating regions of high and low vorticity, indicative of coherent vortex shedding and spanwise modulation. The corrugated configuration promotes localised flow separation and reattachment, generating discrete vortical packets that propagate downstream. These structures are spatially periodic, suggesting a strong influence of the surface geometry on vortex formation and spacing. The normalised vorticity highlights regions of intensified rotational motion, particularly near sharp edges and inflection points, where shear layers roll up into spanwise vortices. The smooth transitions between high and low vorticity zones imply a quasi-steady shedding mechanism, while the three-dimensionality of the surface confirms the presence of spanwise instabilities and secondary flow effects. These results underscore the role of geometric discontinuities in shaping the spanwise coherence and strength of vortical structures, with implications for aerodynamic forces.

### 3.5. Non-Periodic Flow

Non-periodic flow (Re = 10,000) arises when the flow transitions from a regular, time-periodic state (a limit cycle) to a more complex regime where the flow no longer repeats itself in time with a single period, and the frequency spectrum contains several peaks that are not integer multiples of one another. The following sections will discuss specific cases within this flow regime. First we will make a comparison between positive and negative angles of attack, building on the work of [38]. Then, we will consider the transition from 3D non-periodic to periodic following from [23].

#### 3.5.1. Comparison of Negative and Positive *AoA*

**Figure 19:**
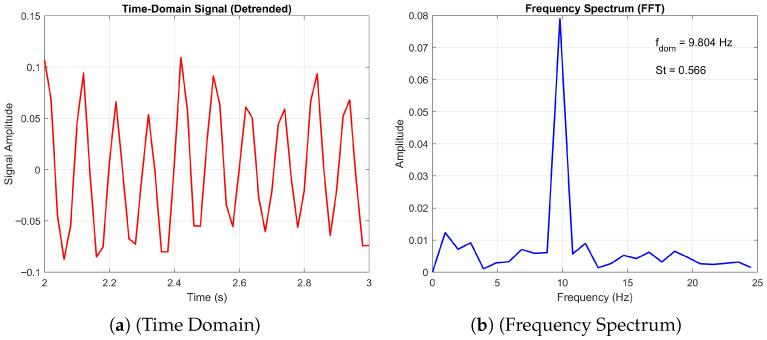
Time-domain signal and frequency-spectrum plots of the corrugated wing at Re=10,000 and AoA = −5°. The red line represents the time-domain signal, and the blue line represents the FFT of the signal.

Figure 19 shows the time-domain signal and frequency spectrum of the corrugated wing at Re = 10,000 and $AoA=-5^{\circ }$, exhibiting a quasi-periodic behaviour with a dominant frequency of $f_{\mathrm{dom}}=9.904\;\mathrm{Hz}$ and a high spectral index (SI=8.566). The spectral index is defined as the ratio of the amplitude at the dominant frequency, $A_{\mathrm{dom}}$, to the mean amplitude of the surrounding spectrum, $\bar{A}$,

(11) $$SI=\frac{A_{\mathrm{dom}}}{\bar{A}},$$

providing a measure of flow coherence. A high spectral index indicates a well-organised flow, where energy is concentrated at the dominant frequency and turbulent fluctuations are minimal, reflecting coherent and stable vortex shedding.

**Figure 20:**
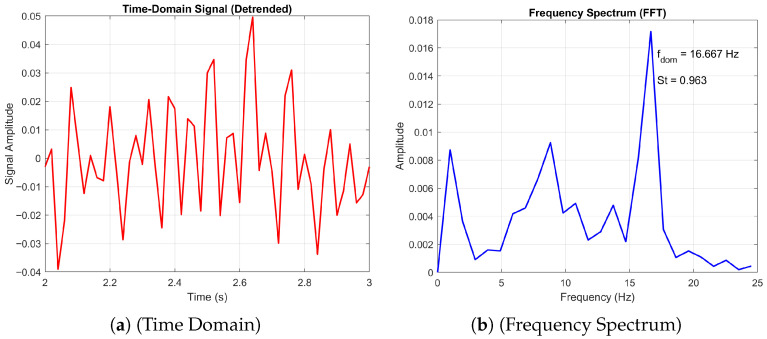
Time domain and frequency spectrum plots of the corrugated wing time domain and frequency plots at Re = 10,000 and *AoA* = 5°. The red line represents the time-domain signal, and the blue line represents the FFT of the signal.

In contrast, Figure 20 presents the response at $AoA=+5^{\circ }$, where the time-domain signal becomes more irregular and the frequency spectrum broadens, with a dominant frequency of $f_{\mathrm{dom}}=16.667\;\mathrm{Hz}$ and a reduced spectral index (SI=3.953). The lower spectral index suggests a less organised flow, consistent with increased turbulence or multi-modal vortex shedding. Together, these results reveal a clear shift in aerodynamic behaviour with angle of attack, highlighting the sensitivity of the corrugated wing’s unsteady flow characteristics to small changes in incidence.

The difference in Strouhal number between the positive and negative angles of attack was presented in the work of [38], where they reported higher Strouhal numbers for positive angles of attack. They stated that for negative angles of attack, the merged vortices stay a longer time in the bigger corrugations, which leads to the reduction in the dominant Strouhal number value compared to negative angles of attack. This observation was confirmed in our simulation, as shown in Table 5.

#### 3.5.2. Transition from Periodic to Non-Periodic

**Figure 21:**
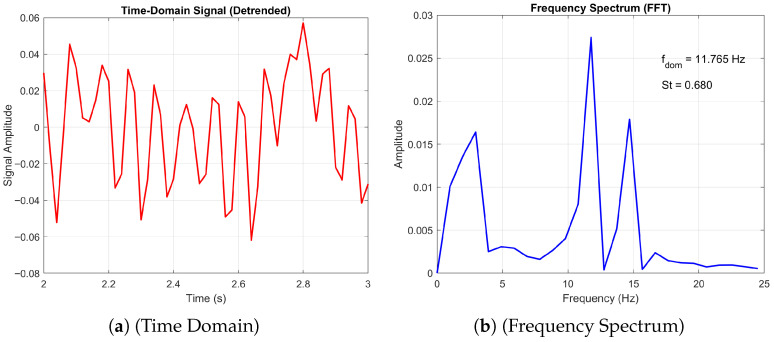
Time domain and frequency spectrum plots of the corrugated wing time domain and frequency plots at Re = 10,000 and *AoA* = 10°. The red line represents the time-domain signal, and the blue line represents the FFT of the signal.

**Figure 22:**
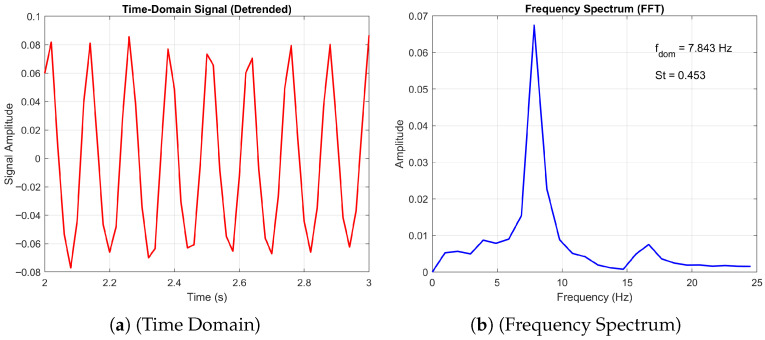
Time domain and frequency spectrum plots of the corrugated wing time domain and frequency plots at Re = 10,000 and *AoA* = 20°. The red line represents the time-domain signal, and the blue line represents the FFT of the signal.

Finally, we consider the transition from a 3D non-periodic to a periodic state. Figure 21 and Figure 22 present the time-domain and frequency spectrum analyses of the aerodynamic signal generated by the corrugated wing at Re = 10,000 for angles of attack $AoA=10^{\circ }$ and $20^{\circ }$, respectively. At $AoA=10^{\circ }$, the flow exhibits a three-dimensional non-periodic character, as evidenced by the irregular fluctuations in the time-domain signal and the broader frequency content. The dominant frequency is identified as $f_{\mathrm{dom}}=11.765\;\mathrm{Hz}$, corresponding to a Strouhal number St=0.690, indicative of smaller-scale, faster unsteady dynamics. In contrast, the signal at $AoA=20^{\circ }$ reveals a transition to a three-dimensional periodic regime, characterised by coherent oscillations in the time domain and a sharper spectral peak at $f_{\mathrm{dom}}=7.843\;\mathrm{Hz}$ (St=0.453). This shift suggests the emergence of organised vortex shedding and larger-scale flow structures at higher incidence, consistent with increased separation and roll-up of the shear layer. The reduction in dominant frequency and Strouhal number reflects the slower, more energetic nature of the periodic flow.

As discussed before, the periodic flow is characterised by a single dominant peak in the frequency domain, as shown for the 20-degree angle of attack. Here we observe a decrease in the Storuhal number as the flow transitions to a periodic state. This was also observed in the DNS of [23], where it was stated that for larger angles of attack, the geometry of the aerofoil produces larger separated flow regions, especially near the trailing edge. These broaden the wake and reduce the strength and interaction rate of shear layers, which leads to slower vortex shedding cycles and, thus, lower Strouhal numbers.

## 4. Conclusions

This numerical investigation of three-dimensional unsteady flow over a bio-inspired corrugated infinite wing revealed several key aerodynamic behaviours across a broad range of Reynolds numbers and angles of attack. At the lowest Reynolds numbers, the flow remained steady and predominantly two-dimensional, with recirculation zones confined to the corrugation valleys. As the Reynolds number increased, the flow transitioned to periodic vortex shedding and the emergence of spanwise three-dimensional structures, confirming that two-dimensional assumptions become unreliable in transitional regimes.

Across the examined conditions, three distinct flow behaviours were identified. In the steady regime, characterised by low Reynolds numbers and small angles of attack, the flow remained laminar and largely two-dimensional, with only localised recirculation and no global instabilities. In the periodic regime, associated with moderate Reynolds numbers or increased incidence, regular vortex shedding produced coherent spanwise structures and oscillatory aerodynamic forces. In the non-periodic regime, corresponding to high Reynolds numbers and transitional conditions, the flow exhibited irregular shedding and turbulent-like features, with broadband spectral content and reduced coherence, resembling bluff body dynamics. This progression illustrates the shift from laminar stability to organised unsteadiness and ultimately to chaotic, non-periodic turbulence.

Our analysis indicates that direct numerical simulation is essential at very low Reynolds numbers, particularly below approximately one thousand, because standard modelling assumptions can introduce substantial errors. Steady flow formulations inherently suppress unsteady mechanisms that are physically present, while stabilisation techniques used in many turbulence models may artificially damp fluctuations, obscure separation behaviour, or modify the onset of vortex shedding. Modelling transitional flows presents additional challenges, as the interaction between laminar and turbulent processes remains a significant open research question and becomes even more complex under varying angles of attack. Although the infinite wing assumption offers a practical and computationally efficient framework, it cannot capture tip effects that strongly influence lift, drag, and the formation of three-dimensional flow structures. These limitations underscore the need for high-fidelity methods, finite span configurations, and parametric spanwise studies in future investigations.

The aerodynamic analysis showed a pronounced reduction in drag between Reynolds numbers of ten and one hundred as inertial effects grew in importance, while lift increased more gradually and began to plateau prior to the onset of turbulence. At a Reynolds number of ten thousand, periodic shedding persisted even at zero incidence, indicating bluff body-like behaviour and marking the emergence of turbulent dynamics. Aerodynamic efficiency reached values comparable to experimental data from dragonfly wings, supporting the reliability of the simulations.

Energy spectrum analysis confirmed the presence of an inertial subrange consistent with classical turbulence scaling, demonstrating that the simulations captured the cascade of turbulent energy. Strouhal number comparisons with wind tunnel measurements showed differences within five to thirteen percent, with improved agreement at higher angles of attack, further validating the numerical predictions.

Overall, the findings show that corrugation does not provide a universal lift-to-drag advantage during steady gliding, but it can delay flow separation in transitional regimes, especially near the root section. The results also highlight the growing influence of spanwise flow structures and identify conditions in which two-dimensional modelling becomes insufficient. This work therefore advances understanding of insect wing aerodynamics and offers guidance for the development of biomimetic wings for low Reynolds number applications, including micro air vehicles.

## Figures and Tables

**Table 1:** A list of simulation parameters used in this study.

Parameter	Value
Chord Length ($L_{c}$)	76 [mm]
Inlet Velocity (*u*)	1.3158 [m/s]
Fluid Density (ρ)	1 [Kg/m^3^]
Dynamic Viscosity (μ)	$1\times 10^{-2}$, $10^{-3}$, $10^{-4}$, $10^{-5}$ [Pa·s]
Time Step (Δt)	0.02 [s]

**Table 2:** Grid convergence study over 4 grids. Data for lift coefficient.

	ϕ	$N_{\mathrm{DoF}}$	*r*	GCI	${\mathrm{GCI}}_{\mathrm{asy}}$	*p*	$\phi_{\mathrm{ext}}$
Grid 1	−1.025 × $10^{-1}$	875,301	1.9	23.83%	0.306	0.97	−1.11 × $10^{-1}$
Grid 2	−9.54 × $10^{-2}$	238,015	2.2	13.66%			
Grid 3	−9.03 × $10^{-2}$	47,801	-	-			
Grid 2	−9.538 × $10^{-2}$	238,015	2.2	32.36%	0.582	0.50	−1.13 × $10^{-1}$
Grid 3	−9.03 × $10^{-2}$	47,801	1.6	28.13%			
Grid 4	−8.82 × $10^{-2}$	19,704	-	-			

**Table 3:** Grid convergence study over 4 grids. Data for drag coefficient.

	ϕ	$N_{\mathrm{DoF}}$	*r*	GCI	${\mathrm{GCI}}_{\mathrm{asy}}$	*p*	$\phi_{\mathrm{ext}}$
Grid 1	1.359 × $10^{-1}$	875,301	1.9	2.10%	0.069	2.00	1.34 × $10^{-1}$
Grid 2	1.42 × $10^{-1}$	238,015	2.2	0.53%			
Grid 3	1.44 × $10^{-1}$	47,801	-	-			
Grid 2	1.420 × $10^{-1}$	238,015	2.2	1.27%	1.401	2.00	1.42 × $10^{-1}$
Grid 3	1.44 × $10^{-1}$	47,801	1.6	8.89%			
Grid 4	1.50 × $10^{-1}$	19,704	-	-			

**Table 4:** Comparison of Strouhal numbers from reference [37] for inclined flat plate and the current study.

*AoA* (°)	*St* [Ref]	*St* [FEM]	Difference (%)
10	0.78	0.68	12.8
20	0.433	0.453	4.42

**Table 5:** Flow regime classification across *AoA* and Reynolds numbers. S = steady, P = periodic, and NP = non-periodic. The number in […] is Strouhal number.

*AoA*/Re	−5	0	5	10	20
10	S	S	S	S	S
100	S	S	S	S	S
1000	S	S	S	2D-P [0.67]	3D-P [0.45]
10,000	3D-NP [0.56]	2D-P [0.03]	3D-NP [0.96]	3D-NP [0.68]	3D-P [0.45]

**Table 6:** Comparison of averaged lift and drag coefficients and lift-to-drag ratios between reference [17] and computed values at Re = 150 and *AoA* = 30.

Quantity	Value	Ref	% Difference
$C_{l}$	1.03	1.06	2.83%
$C_{d}$	0.678	0.759	10.67%
L/D	1.53	1.40	9.29%

**Table 7:** Percentage differences between the 2D simulations of [25] and the current 3D simulations for lift and drag coefficients at various angles of attack.

*AoA* (°)	% Difference in $C_{l}$	% Difference in $C_{d}$
5	9.63%	35.00%
10	6.02%	28.33%
20	4.23%	10.74%

## Data Availability

The data presented in this study are available upon request from the corresponding author.

## Abbreviations

The following abbreviations are used in this manuscript:

2D	Two-dimensional
3D	Three-dimensional
*AoA*	Angle of attack
$C_{d}$	Drag coefficient
$C_{f}$	Skin friction coefficient
$C_{l}$	Lift coefficient
$C_{p}$	Pressure coefficient
CR	Closed recirculation region
DNS	Direct Numerical Simulation
FEM	Finite Element Method
FFT	Fast Fourier Transformation
FR	Free recirculation region
ILES	Implicit Large Eddy Simulation
L/D	Lift-to-drag ratio
LEV	Leading-edge vortex
LSB	Laminar separation bubble
MAV	Micro aerial vehicle
Re	Reynolds number
*St*	Strouhal number
